# New records of Demospongiae (Porifera) from Reserva Marina El Pelado (Santa Elena, Ecuador), with description of *Tedania* (*Tedania*) *ecuadoriensis* sp. nov.

**DOI:** 10.3897/zookeys.1011.54485

**Published:** 2021-01-19

**Authors:** Karla B. Jaramillo, Báslavi Cóndor-Luján, Belinda Longakit, Jenny Rodriguez, Olivier P. Thomas, Grace McCormack, Eduardo Hajdu

**Affiliations:** 1 ESPOL Polytechnic University, Escuela Superior Politécnica del Litoral, ESPOL. Centro Nacional de Acuicultura e Investigaciones Marinas, CENAIM. Campus Gustavo Galindo Km. 30.5 Vía Perimetral, P.O. Box 09-01-5863, Guayaquil, Ecuador; 2 Zoology, School of Natural Sciences and Ryan Institute, National University of Ireland Galway, University Road, H91 TK33 Galway, Ireland; 3 Universidad Científica del Sur, Facultad de Ciencias Veterinarias y Biológicas, Carrera de Biología Marina, Antigua Panamericana Sur Km. 19, Villa El Salvador, Lima, Perú; 4 Marine Biodiscovery, School of Chemistry and Ryan Institute, National University of Ireland Galway, University Road, H91 TK33 Galway, Ireland; 5 Museu Nacional, Universidade Federal do Rio de Janeiro, Depto. Invertebrados, Quinta da Boa Vista, s/n, 20940-040, Rio de Janeiro, RJ, Brazil

**Keywords:** *
Callyspongia
*, *
Cliona
*, sponge taxonomy, Tropical Eastern Pacific

## Abstract

The first taxonomic descriptions of the sponge diversity at El Pelado Marine Protected Area in the province of Santa Elena, Ecuador is reported. Tedania (Tedania) ecuadoriensis Jaramillo & Hajdu, **sp. nov.** is described from its shallow waters. In addition, Callyspongia (Callyspongia) aff. californica (*sensu*Cruz-Barraza and Carballo 2008; *nonsensu*Dickinson 1945) and Cliona aff. euryphylle are reported for the first time. The former species is likely distributed over 4,000 km along the Tropical Eastern Pacific, whereas the latter might be an example of a trans-isthmian lineage. An amended diagnosis for Callyspongia (Callyspongia) and an updated identification key for the subgenera of *Callyspongia* are provided.

## Introduction

Sponges represent a key component of marine ecosystems and exhibit high diversity and abundance in some oceans, including tropical, temperate and polar regions (Bell and Barnes 2003; Bell and Smith 2004; Carballo et al. 2008; Hajdu et al. 2013; Hajdu et al. 2015; Pacheco et al. 2018). Due to the broad substrate cover and filtration capacity of sponges, marine ecosystems in general will likely be affected by changes in the geographic distribution of these organisms (Bell and Smith 2004; Bell 2007; Carballo et al. 2008; Cruz-Barraza and Carballo 2008). These reasons, together with sponges’ known biomedical potential, have attracted the interest of researchers to explore the distribution and diversity of sponges in maritime ecoregions around the planet.

Despite published reports on sponge distribution in the Pacific Ocean, knowledge gaps still exist in the eastern Pacific, and especially along the coast of Ecuador, where descriptive studies have rarely been conducted (Miloslavich et al. 2011). A recent increase in taxonomic effort in this large area started in Chile (Hajdu et al. 2006; Azevedo et al. 2009; Willenz et al. 2009; Hajdu et al. 2013; Fernandez et al. 2016; Costa et al. 2020), and more recently expanded to the Peruvian coast (Aguirre et al. 2011; Azevedo et al. 2015; Hajdu et al. 2015; Cóndor-Luján et al. 2019; Recinos et al. 2020). This collective effort has unveiled a high diversity and abundance of sponges in shallow south-eastern Pacific waters. Since 2003 in Chile, and 2007 in Peru, nearly 3,000 specimens have been collected, with new species reported. Taxonomic identifications of this large collection are still in progress and will certainly lead to many more discoveries.

In this regard, and despite its shorter coastline when compared to Chile and Peru, mainland Ecuador is likely to house a significant diversity of marine sponges. In part, this will be a consequence of being situated in a convergence zone of two different oceanic currents, the northern warm Panama or El Niño Current, and the southern cold Humboldt or Peru Current (Chavez and Brusca 1991; Fiedler et al. 1992; Glynn 2003). In addition, the presence of varied marine coastal ecosystems, such as mangroves, bays, estuaries, and rocky coasts in this area, also supports this hypothesis. So far, knowledge of the sponge biodiversity of Ecuador is entirely restricted to descriptions of species from the Galapagos Islands (Desqueyroux-Faúndez and van Soest 1997; Bustamante et al. 2002). According to the World Porifera Database (WPD), 87 species have been recorded from these islands (van Soest et al. 2020), both from the shallow waters (Topsent 1895; Wilson 1904; Lendenfeld 1910; de Laubenfels 1939; Lee 1987; Hajdu and Desqueyroux-Faúndez 1994; Desqueyroux-Faúndez and van Soest 1996, 1997; Sarà et al. 2000) and deeper waters (Schuster et al. 2018). Surprisingly, no taxonomic study focused on the sponge diversity of coastal mainland Ecuador, a gap due to a historical lack of baseline scientific initiatives and sponge taxonomists in this region. In order to start reverting this scenario, a national project was funded by the Ecuadorian government aiming at the description of the marine invertebrate biodiversity, and their associated chemical and microbial diversity, in a small marine protected area of the peninsula of Santa Elena, named El Pelado Marine Reserve (REMAPE). The first results of this biodiversity assessment have recently been published with a focus on zoantharians (Jaramillo et al. 2018), and sponges come next, also selected for their known content of metabolites with potential pharmacological use (Carroll et al. 2019). The goal of the present study was therefore to describe the most abundant species of sponges occurring in the El Pelado MPA. Herein, we present three new records of Demospongiae for this area, including one new species.

## Materials and methods

### Biological material

**Figure 1.**
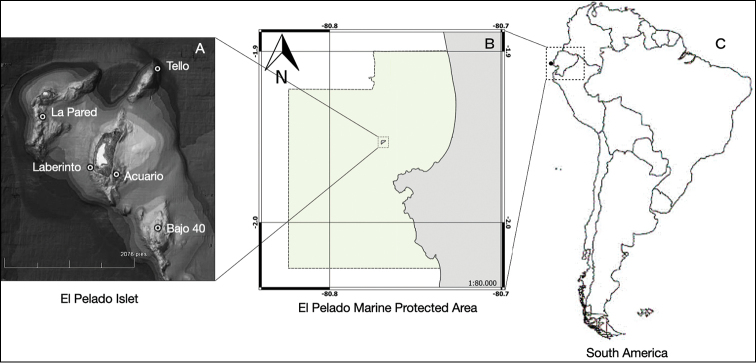
Map of the Marine Protected Area El Pelado **A** The Pelado islet and its submarine platform with five sampling locations of the sponges **B** map of the whole Marine Protected Area El Pelado at the Ecuadorian coast **C** map of South America highlighting Ecuador (map created using QGIS software Version 3.2).

Specimens were collected using SCUBA diving in different sites of the El Pelado Marine Reserve (REMAPE – Santa Elena, Ecuador, -80.8221, -1.9228) and photographed in situ (Fig. 1). Samples were preserved in 95% EtOH and a voucher sample of each species is deposited at the scientific reference collection of Centro Nacional de Acuicultura e Investigaciones Marinas (**CENAIM**), with some fragments shared with the sponge collections at Museu Nacional do Rio de Janeiro (**MNRJ**) /Universidade Federal do Rio de Janeiro (**UFRJ**, Rio de Janeiro, Brazil).

### Morphological examination

Species identification and morphological descriptions were achieved from ethanol samples and in situ observations. Descriptions were based on dissociated spicules and thick anatomical sections obtained for each specimen following standard protocols outlined in Hajdu et al. (2011). Dissociated spicules and thick sections slides were examined under an EVOS Digital Colour Fluorescence Microscope. For each spicule category, at least 20 measurements were made. Metrical data are given in micrometres, unless otherwise indicated, as the range, with the mean and the number of measurements taken (n) in parentheses. The Scanning Electron Microscopes (SEMs) used to obtain the spicule electron micrographs were a JEOL 6390LV at Museu Nacional (UFRJ), and a Hitachi S-4700 SEM at National University of Ireland, Galway. The classification adopted here is that of the ‘Systema Porifera’ (Hooper and van Soest 2002), as modified by Morrow and Cárdenas (2015), and implemented in the World Porifera Database (van Soest et al. 2020).

## Results

### Systematics

#### Phylum Porifera Grant, 1835

**Class Demospongiae Sollas, 1885**

**Subclass Heteroscleromorpha Cárdenas, Pérez & Boury-Esnault, 2012**

**Order Poecilosclerida Topsent, 1928**

**Family Tedaniidae Ridley & Dendy, 1886**

**Genus *Tedania* Gray, 1867**

**Subgenus Tedania Gray, 1867 *sensu*Aguilar-Camacho et al. (2018)**

##### Tedania (Tedania) ecuadoriensis

Taxon classificationAnimaliaPoeciloscleridaTedaniidae

Jaramillo & Hajdu sp. nov.

27D26847-725D-51C3-AA01-05752B3F8CD3

http://zoobank.org/05C00AF3-E75C-402E-AAA1-2D511771ADEF

Figure 2A–I

###### Diagnosis.

Tedania (Tedania) with rather small ectosomal tylotes (139–185 µm), and choanosomal styles (127–183 µm), as well as possessing two size categories of onychaetes (71–133 and 29–69 µm).

###### Etymology.

Named after the country where its type locality is situated.

###### Type material.

***Holotype***: CENAIM 150813EP01-05 with fragment as MNRJ 19918, El Pelado Islet (‘La Pared’, -1.932847; -80.792453), REMAPE, Santa Elena, Ecuador, 13 m deep, collected by O. Thomas, 13 Aug. 2015. ***Paratype***: CENAIM 150825EP04-05 with fragment as MNRJ 19923, El Pelado Islet (‘Laberinto’, -1.9355; -80.7896), REMAPE, Santa Elena, Ecuador, 5 m deep, collected by K. Jaramillo, 25 Aug. 2015.

###### Habit

**Figure 2.**
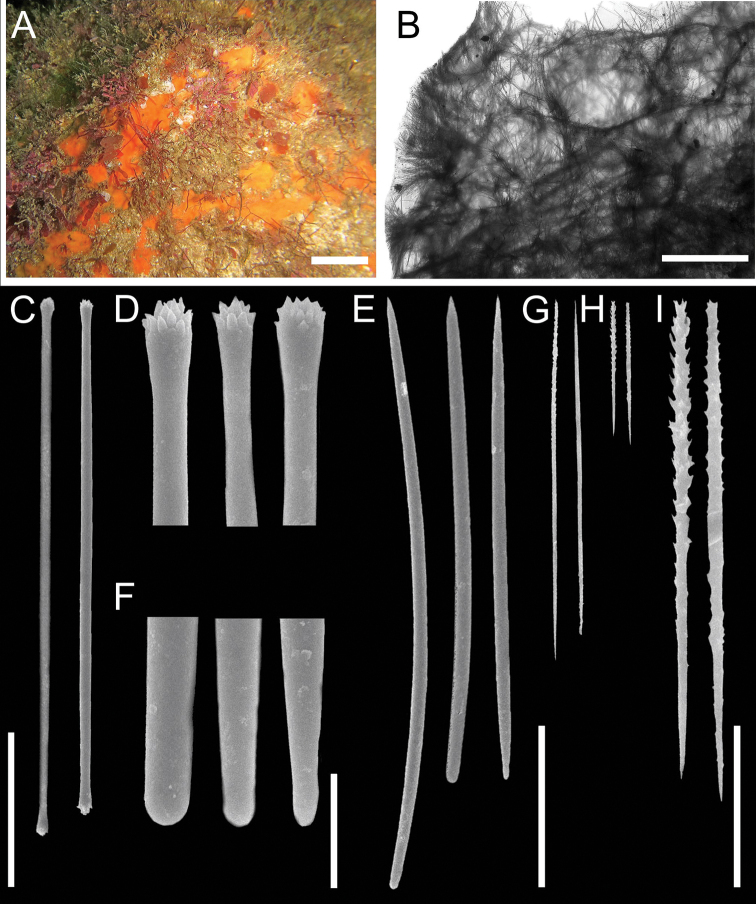
Tedania (Tedania) ecuadoriensis sp. nov. **A** holotype in situ (CENAIM 150813EP01-05) **B** transverse section of ectosomal and choanosomal skeletal architecture **C** ectosomal tylotes **D** detail of terminally microspined terminations of ectosomal tylotes **E** choanosomal styles **F** detail of the bases of choanosomal styles **G** large onychaetes **H–I** small onychaetes. Scale-bars: 2 cm (**A**); 500 µm (**B**); 50 µm (**C, E, G–H**); 10 µm (**D, F, I**).

(Fig. 2A). Thickly encrusting to massive (thickness: 0.3 cm). The holotype is fragmented, and the largest piece measures 0.8 × 0.4 cm. Oscula located at the top of short elevations. Consistency soft and compressible. Texture smooth. Colour in life is orange, and in ethanol it turns to violet with some red spots on the surface.

###### Skeleton

(Fig. 2B). Ectosomal architecture with brushes of tylotes, some of which pierce the surface, not easily detachable from the choanosome. Choanosomal architecture a dense, confused reticulation of styles and scattered onychaetes.

###### Spicules.

Megascleres (Fig. 2C–F, Table 1): Ectosomal tylotes, 139–85 (168; n = 30); choanosomal styles, 127–183 (155; n = 35). Microscleres (Fig. 2G–I): larger onychaetes, 71–133 (92; n = 41); smaller onychaetes, 29–69 (41; n = 25).

**Table 1.** Morphology of spicules, locality and depth, for East Pacific *Tedania* spp. seemingly closer to T. (T.) ecuadoriensis sp. nov., and *T. ignis*. Spicule morphometrics are in micrometres as range with the mean in parentheses, n.r. is not reported.

Species	Tylotes	Styles	Onychaetes	Locality / depth
*T. ecuadoriensis* sp. nov.	139–185 (168) × 1.9–3.6 (2.4)	127–183 (155) × 2.1–5.5 (3.8)	I, 71–133 (92)	El Pelado Islet / 5–13 m
			II, 29–69 (41)	
*T. fulvum* (Aguilar-Camacho et al., 2018) (orig. descr.)	130–150 (142.5) × 2.5–5 (2.7)	135–185 (171.5) × 2.5–5 (3.4)	30–120 (60.5) × 0.5–1	Mexican Pacific / 8 m
*T. galapagensis* (Desqueyroux-Faúndez & van Soest, 1996) (orig. descr.)	179–234 (198) × 3	192–246 (226) × 6	I, 173–205 (188) × 2	Galapagos / 78 m
			II, 61–93 (78) × 0.5–1	
*T. obscurata* (de Laubenfels, 1930) (orig. descr.)	200–300 × 6–12	present, but rare	I, n.r.	California / intertidal
			II, 80 × 2	
*T. tepitootehenuaensis* (Desqueyroux-Faúndez, 1990)(orig. descr.)	192–250 (227) × 3–7 (5)	204–272 (241) × 4–9 (7)	I, 160–285 (188) × 2–3 (2)	Easter Isl. / 3 m
			II, 48–76 (59) × 0.5–0.9 (0.6)	
*T. topsenti* (de Laubenfels, 1930) (orig. descr.)	200 × 8	250 × 11	I (?), 180 × 2 reported as “? raphides”, and suggested must be young megascleres instead	California / intertidal
*T. toxicalis* (de Laubenfels, 1930) (orig. descr.)	200 × 8–14	100–200 × 2–7	I, 150	California / intertidal
			II, “not observed”	
*T. tropicalis* (Aguilar-Camacho et al., 2018) (orig. descr.)	150–210 × 2.5–5	150–215 × 2.5–7.5	I, 90–180 × 0.5–1.8	Mexican Pacific / 1–5 m
*T. ignis* (Duchassaing & Michelotti, 1864) sensu Zea (1987)	181–242 (220.4) × 3–6 (4.3)	228–313 (259.8) × 3–8 (4.3)	I, 142–280 (230.7) × 1–3 (1.9)	Tropical W Atlantic / 0–3 m
			II, 52–138 (83.3) × 0.5–2 (1.2)	

###### Ecology and distribution.

The species was found between 5–13 m depth, close to red algae, and slightly covered with sediment. No dermatitis reaction was observed after contact with bare skin.

###### Remarks.

Six species of *Tedania* were described from the Tropical Eastern Pacific (de Laubenfels 1930; Desqueyroux-Faúndez 1990; Desqueyroux-Faúndez and van Soest 1996; Desqueyroux-Faúndez and van Soest 1997; Aguilar-Camacho et al. 2018), namely *T. fulvum* Aguilar-Camacho, Carballo & Cruz-Barraza, 2018; *T. galapagensis* Desqueyroux-Faúndez & van Soest, 1996; *T. obscurata* (de Laubenfels, 1930); *T. tepitootehenuaensis* Desqueyroux-Faúndez, 1990; *T. topsenti* de Laubenfels, 1930; *T. toxicalis* de Laubenfels, 1930 and *T. tropicalis* Aguilar-Camacho, Carballo & Cruz-Barraza, 2018. *Tedania galapagensis* was an obvious first hypothesis for the identification of the El Pelado *Tedania*, for its occurrence in the relatively nearby Galapagos Archipelago, but we found it to be distinct from the new species by its tylotes, styles and onychaetes, with much larger dimensions than observed in our new species. When variation of this sort occurs intraspecifically, it is the continental specimen to harbour the largest spicules, as a consequence of likely increased levels of dissolved silica in comparison to oceanic locations (for a discussion in the context of the Caribbean, see Zea (1987). Furthermore, *T. galapagensis* was reported from deeper waters (78 m) than those where *T. ecuadoriensis* sp. nov. was found (5–13 m). The species appearing closest to our material, as far as spicule micrometrics go, is the Mexican *T. fulvum*, that also has a set of relatively small spicules. The onychaetes, reported in a single, variable size-category, match nearly perfectly the full range observed in both categories combined of the new species. On the other hand, the coelosphaerid/hymedesmiid-kind of ectosomal tylote, with smooth, pronounced, elliptical heads, finds no match in the new species, and is seen here as decisive evidence of the non-conspecificity of both species.

*Tedania ecuadoriensis* sp. nov. with its short tylotes (168 µm, mean length) and styles (155 µm, mean length) is distinct from all known (sub)Tropical Eastern Pacific *Tedania* spp. which have these around 200 µm or bigger. *Tedania ignis*, a Tropical Western Atlantic species with considerable overall similarity to the new species proposed, also bears much larger megascleres and microscleres, which contradicts any hypothesis of possible conspecificity.

#### Order Clionaida Morrow & Cárdenas, 2015

**Family Clionaidae d’Orbigny, 1851**

**Genus *Cliona* Grant, 1826**

##### Cliona aff. euryphylle

Taxon classificationAnimaliaClionaidaClionaidae

Topsent, 1888

8A3876A3-EA7C-5592-A81A-2E4A7BFF0729

Figure 3A–E

###### Material examined.

CENAIM: 160510EP07-01, El Pelado Islet (‘Bajo 40’, -1.938217; -80.786669), REMAPE, Santa Elena, Ecuador, 12 m deep, collected by K. Jaramillo, 10 May 2016.

###### Habit

**Figure 3.**
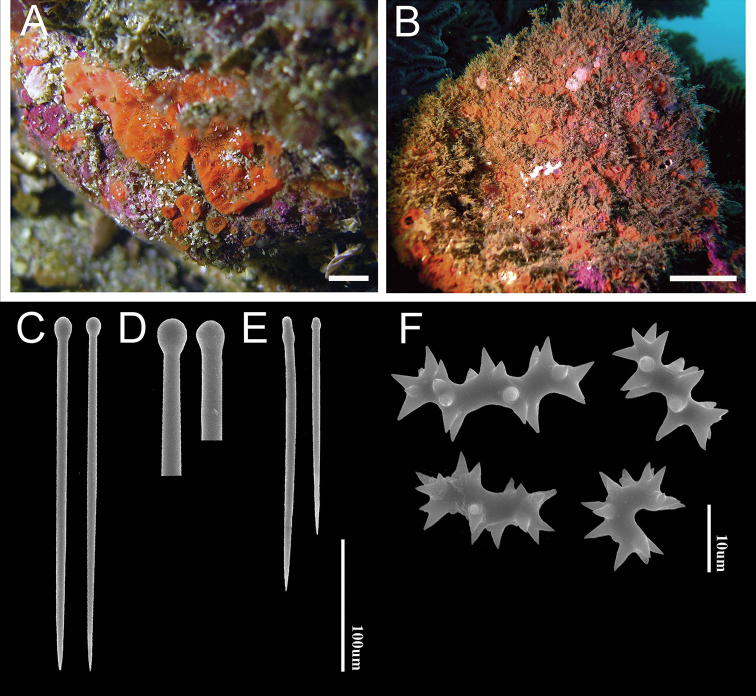
Cliona aff. euryphylle Topsent, 1888 **A, B** specimen alive in situ (CENAIM 160510EP07-01) collected at El Pelado Marine Reserve **C** large tylostyles **D** heads of tylostyles **E** small tylostyles **F** different sizes of spirasters with large spines. Scale-bars: 1 cm (**A**); 5 cm (**B**); 100 µm (**C–E**); 10 µm (**F**).

(Fig. 3A, B). Encrusting alpha stage over 15 × 15 cm in area. Extended papillae with slightly elevated (up to 5 mm) oscula, up to 2 mm in diameter. Sponges form small patches, with a firm texture, even after removal from substrate. The colour in life is orange, turning to pale yellow in ethanol.

###### Skeleton.

Typical *Cliona* arrangement, with an ectosomal palisade of tylostyles, and the same spicules in a confused arrangement in the choanosome. Small spirasters are scarce in the papillae, but they occur abundantly dispersed in the choanosome.

###### Spicules.

Megascleres (Fig. 3C–E, Table 2). Tylostyles, 221–336 (267; n = 34) × 5–11 (7, n = 30), with pronounced rounded to oval heads. Microscleres (Fig. 3F). Small and robust spirasters 8–35 (19; n = 32) × 3–8 (5; n = 27), with several large conical spines spiralling around the shaft, in helical and S-shaped forms; occasionally approaching amphiasters morphology.

**Table 2.** *Cliona euryphylle*Topsent 1888 and Cliona aff. euryphylle: Morphology of spicules, locality and depth for specimens studied here, and from the literature. Species deemed more closely related are included for comparison. Spicule morphometrics are in micrometres as range with the mean in parentheses, n.r. is not reported.

Species	Tylotes	Spirasters	Locality / depth
C. aff. euryphylle	I, 221–336 (267) × 5–11 (7);	8–35 (19) × 3–8 (5)	El Pelado Islet / 5–10 m
	II, 115–264 (211) × 5–9 (7)		
*C. euryphylle* (Topsent, 1888) (orig. descr.)	300 × 5	35 × 5	Southern Gulf of Mexico
sensu de Laubenfels (1954)	300 × 7	n.r. × 4–8	Micronesia / 5 m
sensu Bergquist (1968)	290–392 (344) × 9.5–17.5 (12.5)	7–28 (24) × 0.9–9.2 (6.3)	New Zealand / 25 m
sensu Carballo et al. (2004)	180–367.5 (277) × 2.5–10 (5.5)	10–30 (18.1)	Mexican Pacific / 4–20 m
sensu Vega (2012)	111–365 × 1.3–11	30–6	Mexican Pacific / 0–3 m
sensu Pacheco et al. (2018)	120–300 (201) × 5–8 (6.7)	9–24 (18) × 2–7 (4.7)	Costa Rica Pacific / 4–20 m
*C. aethiopicus* (Burton, 1932) (orig. descr.)	260 × 7	28	Gulf of Guinea / 18–30 m
*C. burtoni* (Topsent, 1932) (orig. descr.)	225–330 (175) × 7–12 (2.5)	15–28 (40) × 5–6 (1.5)	Mediterranean / N/A
sensu Bertolino et al. (2013)	132–287 (225) × 5–7.5 (6)	10–45 (26.5) × 1.3–17.5 (10)	Mediterranean / 30 m
*C. caledoniae* (van Soest & Beglinger, 2009) (orig. descr.)	246–426 (360.9) × 8–12 (9.8)	19–31 (24.3) × 5–9 (6.8)	NE Atlantic / 82–131 m
*C. dioryssa* (de Laubenfels, 1950); sensu Rützler (1974)	107–392 (244.4) × 3.7–7.4 (5.4)	I, 11–42 (27.4) × 1.4–4.8 (3.2) (shaft);	Bermuda / 0–12 m
		II, 19–43 (33.9) × 0.6–2.2 (1.5) (shaft)	
sensu Muricy and Hajdu (2006)	200–440	I, 25–40;	SE and NE Brazil / 5–25 m
		II, 10–20	

###### Ecology and distribution.

Occurs from 5 to 10 m depth, over rocks, excavating shells, near red and brown algae, and slightly surrounded with sediment. *Cliona euryphylle* Topsent, 1888 was originally described from the Atlantic Ocean (Gulf of Mexico) by Topsent (1888), followed by a series of records from the Pacific: de Laubenfels (1954) in the Central Pacific, Bergquist (1968) in New Zealand; Carballo et al. (2004); Carballo et al. (2008); Vega (2012) in the Mexican Pacific; and Pacheco et al. (2018) in the Costa Rican Pacific.

###### Remarks.

Our preliminary results are inconclusive with regard to the identification of this Ecuadorian *Cliona* material, as no DNA sequence has been published for *C. euryphylle*, let alone for an Atlantic record of the species. It is also possible that the Ecuadorian species might belong to a distinct species, rather than suppose its crossing of the isthmus through the Panama Canal, as explicitly suggested by Pacheco et al. (2018). Previous records of *C. euryphylle* need to be revised in an integrative approach with more extensive sampling and molecular analyses with higher resolution capabilities.

Meanwhile, we can highlight what these populations share and what distinguishes them from one another in morphological terms. The first, but unlikely, biogeographical record of *C. euryphylle* is that by de Laubenfels (1954) from Micronesia, perhaps misled by his mistaken interpretation of Topsents’ type locality, assumed to be in the Eastern Pacific (Topsent 1888). Even though the proposed transpacific track is unlikely, de Laubenfels’ brief description hampers further discussion without re-examining this material. Likely misguided by de Laubenfels’ pioneering transpacific range extension, Bergquist (1968) registered the species from New Zealand shallow waters. This is another unlikely record simply from its distance from previous localities. Furthermore, Bergquist offered some observations that might be interpreted to be suggestive of non-conspecificity, such as the larger dimensions of megascleres (up to 392 µm), and the abundance of microscleres. The tylostyles in the specimens described by Bergquist (1968) were reported to reach 17.5 µm in thickness, while Topsent’s original data indicates 5 µm. The same applies to the thickness of the spirasters in Bergquist’s specimens (≤ 9 µm thick, or ≤ 14 µm, if spines are included), while Topsent mentioned a thickness of 5 µm. These differences indicate that these populations do not belong to the same species.

However, a series of records exists that have been considered indicative of the species’ transisthmian distribution (Carballo et al. 2004; Vega 2012; Pacheco et al. 2018). These report on sponges bearing tylostyles up to 368 µm long, and 11 µm thick (Carballo et al. 2004; Vega 2012) respectively, but also, in the case of Pacific Costa Rican specimens, only up to 300 × 8 µm (Pacheco et al. 2018), which considerably approach values originally reported by Topsent. On the other hand, spirasters appear to fall short from those of Topsent, up to nearly 50% longer. While the possibility cannot be discarded that these amphi-American populations belong to the same species, this should be verified by an alternative dataset, as suggested above.

Cliona aff. euryphylle shares the same spicules (thick and short spirasters) with four other *Cliona* spp., namely *C. aethiopicus* Burton, 1932, *C. burtoni* Topsent, 1932, *C. caledoniae* van Soest & Beglinger, 2009 and *C. dioryssa* (de Laubenfels, 1950). However, these species have unusual aspects of their spirasters, both in dimensions as well as outline, which suggest closer proximity between the Ecuadorian species and *C. euryphylle*. *Cliona aethiopicus* was considered closely allied to *C. chilensis* by Burton (1932), irrespective of Thiele’s (1905) hesitation regarding the origin of a few spirasters found in the encrusting Chilean specimen he studied. The presence of these spirasters in the type material of *C. chilensis* was not confirmed by Desqueyroux-Faúndez and van Soest (1997), which establishes both species’ spicule sets as markedly divergent. The former, with abundant microscleres. The latter, devoid of those. Contrastingly to what we have observed in the Ecuadorian C. aff. euryphylle, with varied microsclere morphologies, Burton (1932) did not mention any variation in the spirasters of *C. aethiopicus*.

*Cliona burtoni* has spirasters with proportionately much shorter spines, and much straighter axes when compared to the pattern seen in C. aff. euryphylle. Furthermore, the tylostyles with predominantly subterminal heads present in *C. burtoni*, are only occasionally present in the latter species. *Cliona caledoniae* has spirasters bearing extremely stout and somewhat obtuse spines that differ considerably from the pointier spines seen in C. aff. euryphylle. Finally, *C. dioryssa*’s tylostyles approach 400 µm, and the species has two categories of spirasters of rather varied morphology, reaching over 40 µm in length, also appearing distinct from those in C. aff. euryphylle.

#### Order Haplosclerida Topsent, 1928

**Family Callyspongiidae de Laubenfels, 1936**

**Genus *Callyspongia* Duchassaing & Michelotti, 1864**

##### Subgenus Callyspongia

Taxon classificationAnimaliaHaploscleridaCallyspongiidae

Duchassaing & Michelotti, 1864

DAFA6598-D81E-5B19-B2D3-D5A6C9FF2A03

###### Diagnosis.

*Callyspongia* with smooth surface, ectosomal skeleton not echinated, spongin sheath conspicuous, no fibrofascicles. Modified from (Desqueyroux-Faúndez and Valentine 2002).

###### Remarks.

The emphasis by Desqueyroux-Faúndez and Valentine (2002) on a single size of ectosomal mesh of “regular size of single, rounded to polygonal mesh”, in their own words was misleading, as a hierarchical pattern of smaller meshes within larger meshes is apparent in several morphological descriptions of the type species, *C. fallax* (Duchassaing & Michelotti, 1864), such as those by van Soest (1980) and Zea (1987).

### Identification key to the subgenera of *Callyspongia*

**Table:** 

1	Ectosomal skeleton echinated	**2**
–	Ectosomal skeleton not echinated	**3**
2	Ectosomal echination by a strong palisade of spicule brushes; narrow spongin sheath on primary multi-spicular choanosomal fibres	*** Cavochalina ***
–	Ectosomal echination by free spicules; large spongin sheath on primary paucispicular choanosomal fibres	*** Euplacella ***
3	Surface smooth; spongin sheath conspicuous	*** Callyspongia ***
–	Surface conulose to spiny; spongin sheath only seldom conspicuous, mostly meagre or absent	**4**
4	Spongin always visible; toxas absent	*** Cladochalina ***
–	Spongin scarce; toxas always present	*** Toxochalina ***

#### Callyspongia (Callyspongia) aff.californica

Taxon classificationAnimaliaHaploscleridaCallyspongiidae

Dickinson, 1945

ED350A1A-CEB9-5F3C-91F1-6721E471B243

sensu Cruz-Barraza and Carballo (2008) Figure 4A–H

Callyspongia cf. californica – Calabro et al. 2018: supp. inf., P3.

##### Material examined.

CENAIM 150820EP02–01 with fragment as MNRJ 19920, ‘Acuario’, El Pelado Islet, REMAPE, Santa Elena, Ecuador (-1.936167; -80.788922), 6 m deep, collected by K. Jaramillo, 20 Aug. 2015. CENAIM 150813EP07–07 or MNRJ 19924, ‘Bajo 40’, El Pelado Islet, REMAPE, Santa Elena, Ecuador (-1.938217; -80.786669), 15 m deep, collected by O. Thomas, 13 Aug. 2015. CENAIM 150825EP04–04 or MNRJ 19925, ‘Laberinto’, El Pelado Islet, REMAPE, Santa Elena, Ecuador (-1.9355; -80.7896), 5 m deep, collected by K. Jaramillo, 25 Aug. 2015 [voucher from Calabro et al. 2018]. CENAIM 160213EP04–01 or MNRJ 19949 and CENAIM 160213EP04–02 or MNRJ 19951, ‘Laberinto’, El Pelado Islet, REMAPE, Santa Elena, Ecuador (-1.9355; -80.7896), 5–7 m deep, collected by O. Thomas, 13 Feb. 2016.

##### Material studied for comparison.

*C. californica*, voucher number: IRCSET364 from Parque de la Reina Acapulco, Acapulco, México (16.8491314; -99.9015755), 4–15 m deep, collected by J.L. Carballo, 01 Jul. 2012. Molecular Evolution and Systematics (MEAS) collection at National University of Ireland, Galway (NUIG).

##### Habit

**Figure 4.**
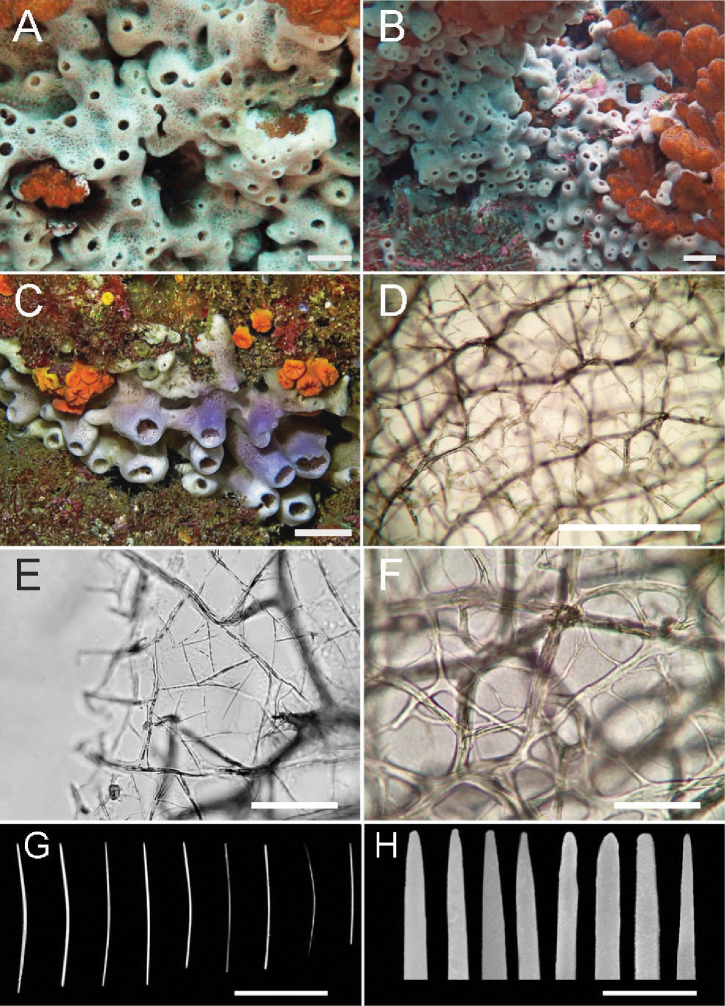
Callyspongia (Callyspongia) aff. californica Dickinson, 1945 (*sensu*Cruz-Barraza and Carballo 2008) **A–C** specimens alive in situ: **A**CENAIM 150820EP02-01 **B**CENAIM 150813EP07–07 **C** specimen collected for chemical studies CENAIM 150825EP04-04, of the rarer tubular morphotype **D** delicate ectosomal reticulation seen through stouter subectosomal polygonal meshes **E** detail of ectosomal reticulation with primary and secondary meshes **F** detail of subectosomal reticulation showing stouter, multi-spicular tracts **G** oxeas **H** details of the terminations of the oxeas. Scale bars: 1.5 cm (**A–C**); 1000 µm (**D**); 400 µm (**E–F**); 50 µm (**G**); 50 µm (**H**).

(Fig. 4A–C). Cushion-shaped, usually up to 2–3 cm thick only, frequently bearing short irregularly cylindrical or volcano-shaped projections topped by roundish oscula, 1–5 mm in diameter. Occasionally, larger coalescent tubes with apical oscula (over 1 cm in diameter) can also be seen. Consistency soft, easily torn, yielding moderate amounts of mucus upon collection and handling. Texture smooth. Colour in life ranging from white to blueish/purplish, becoming beige in ethanol.

##### Skeleton

(Fig. 4D–F). Ectosomal architecture a neat reticulation of polygonal primary and secondary meshes, the former outlined by pauci- to multi-spicular fibres, the latter by thin very slender fibres, mostly one or two spicules across, and a single spicule long. Choanosomal architecture an irregular polygonal reticulation of pauci- to multi-spicular fibres, frequently sinuous.

##### Spicules

(Fig. 4G–H, Table 3). Oxeas in a single size category, slender, slightly irregular, mostly slightly curved or bent in the middle, mostly with slightly roundish, irregular ends, 46–83 (57, n = 31) × 1.2–5.0 µm (2.5, n = 28).

**Table 3.** Callyspongia (C.) californica Dickinson, 1945 and C. (C.) aff. californica: Morphology of spicules spicule (in micrometres), locality and depth for specimens studied here, and from the literature. Spicule morphometrics are in micrometres as range with the mean in parentheses.

Species	Oxeas	Locality / depth
C. aff. californica	46–71 (57.5) (N = 30)	El Pelado Islet / 6 m
150820EP02–01		
(MNRJ 19920)		
150813EP07–07	48–69 (61.2) (N = 30)	El Pelado Islet /15 m
(MNRJ 19924)		
150825EP04–04	50–81 (67.3) (N = 13)	El Pelado Islet / 5 m
(MNRJ 19925)		
160213EP04–01	61–74 (65.9) (N = 16)	El Pelado Islet / 7 m
(MNRJ 19949)		
160213EP04–02	50–83 (63.9) (N = 06)	El Pelado Islet / 5 m
(MNRJ 19951)		
IRCSET364	56–105 (67) × 1.5–5.0 (2.4)	Mexican Pacific / 8 m
*C. californica* Dickinson, 1945 (orig. descr.)	80–150 × 3–5	Mexican Pacific / beached (“shore”)
sensu Sim and Bakus (1986)	84–132 (105) × 2.4–7 (5.3)	California / 3.6 m
sensu Cruz–Barraza and Carballo (2008)	52–117 (73) × 1.3–5.0 (2.4)	Mexican Pacific / 0–15 m

##### Ecology and distribution.

The sponge is quite abundant in the shallow waters at El Pelado and in the nearby continental shore, where it occurs in areas of considerable water flush, frequently in close association with *Pocillopora* (Lamarck, 1816) corals and many species of octocorals. Red algal turfs are frequently seen as epibionts. Callyspongia aff. californica also presents a complete family of bioactive amphiphilic compounds named callyspongidic acids that have inhibitory properties against the melanoma cell line A2058, metabolites that could be important for further chemotaxonomy studies (Calabro et al. 2018). Its recorded depth at El Pelado is 5–20 m, but its frequent finding beached after storms suggests its occurrence in even shallower waters. This might be the southernmost record of *C. californica* (*sensu*Cruz-Barraza and Carballo 2008; *nonsensu* Dickinson, 1945), formerly known only from Mexico (Cruz-Barraza and Carballo 2008) and possibly California (Sim and Bakus 1986), and first time citation to the Tropical South-eastern Pacific (but see the Remarks section). Interestingly, *C. californica* has also been observed in close association with *Pocillopora* corals in the Mexican Pacific coast.

##### Remarks.

*Callyspongia californica*, originally reported from Mexico (Dickinson 1945), has subsequent records from California (Sim and Bakus 1986) and several Mexican locations (Cruz-Barraza and Carballo 2008). Dickinson (1945) highlighted the 150 µm long oxeas in his *C. californica* material as the most striking feature separating this species from any other. Sim and Bakus (1986) found oxeas only up to about 130 µm in California. Then, Cruz-Barraza and Carballo (2008), in spite of studying nearly topotypical specimens (those from Oaxaca), could not find oxeas larger than 117 µm. We had access to a comparative sample kindly sent on loan by JL Carballo, obtained from Acapulco (Guerrero), only about 400 km distant from the type locality at Tangola Island (Oaxaca). While spicule dimensions in the Ecuadorian specimens are even smaller, being ≤ 100 µm in the specimens sampled, the ability of *C. californica* to build oxeas of different sizes even in the same locality (JL Carballo pers. comm. 2019) suggests that spicule size is not a useful trait to distinguish the Ecuadorian specimens as a separate species. Despite the observed difference in spicule dimensions, overall morphological and ecological similarity strongly indicate that our specimen from Ecuador is most likely conspecific to the Mexican sample studied.

Calabro et al. (2018) provided a brief description of the Ecuadorian species in their Supporting Information, but the rationale for this name choice is only given here. Had we revised the species’ type specimen, and generated sequences for faster evolving markers, we might have been confident of Ecuadorian specimens being best assigned to *C. californica*, despite a Meridional occurrence about 4,000 km distant from the species’ previously known geographic range. Unfortunately, the type specimen could not be located and may have been lost/misplaced in the transfer of the Dickinson material from the University of Southern California to the Natural History Museum of Los Angeles (K. Omura, pers. comm. May 31^st^, 2019), so we could not attempt to extract DNA from it for comparison. As such, we opted to identify the Ecuadorian species as C. aff. californica instead, for the time being. Whether subsequent Mexican records of *C. californica* (e.g., Carballo et al. 2004, 2008; Vega 2012) are indeed conspecific with the type specimen of this species, we cannot say. Conservatively, we suggest them being provisionally best referred to as C. aff. californica too.

## Discussion

This work represents the first morphological study of sponges off the coast of mainland Ecuador. As a result, we revealed two new records for the Ecuadorian mainland coast, and the South-eastern Pacific, and one new species *Tedania ecuadoriensis* sp. nov., provisionally endemic of the MPA El Pelado, at the Guayaquil Marine Ecoregion, Tropical Eastern Pacific Realm.

Information and interest in Ecuadorian sponges have increased in recent years as a consequence of the growing body of evidence on the marked underestimation of sponge biodiversity in the whole SE Pacific (e.g., Azevedo et al. 2009, 2015; Hajdu et al. 2013). Increasingly, this renewed interest in the biodiversity inventory of this broad area has been conducted via an integrative approach, by combining classical morphological techniques with an assessment of several molecular markers (De Paula et al. 2012; Azevedo et al. 2015). This will be the way to progress if a sound increase in knowledge on Ecuadorian marine sponges is sought. This is made clear by the partial identifications put forward here for Callyspongia aff. californica and Cliona aff. euryphylle. The final conclusive identification of these species depends on the integration of alternative data sources, which has not been possible here for several reasons: missing type materials, lack of well-preserved topotypical specimens, failure to obtain sequences from the ideal molecular markers, and time constraints.

The hesitant species determination provided here for C. aff. euryphylle and C. aff. californica derive in the first place from the insufficient data available for the type specimens of both species. The lack of type data meant that despite how similar Ecuadorian data was to more recent comprehensive records of both species, we could not conclusively identify both. Only molecular evidence can confirm these possibly discontinuous distributions, as verified for *C. celata* Grant, 1826 in the Western Atlantic (De Paula et al. 2012; Gastaldi et al. 2018), or the calcareous sponge *Clathrina aurea* Solé-Cava, Klautau, Boury-Esnault, Borojevic & Thorpe, 1991, formerly endemic to Brazil, but recently recorded from Peru (Azevedo et al. 2015) and the Caribbean (Fontana et al. 2018). Whether subsequent Mexican records of *C. californica* (e.g., Carballo et al. 2004, 2008; Vega 2012) are indeed conspecific with the type specimen of this species, we cannot say. We feel confident though, that the Ecuadorian specimens are conspecific to these subsequent Mexican records of *C. californica*, all of which we suggest being provisionally best referred to as C. aff. californica.

Despite the value of the integrative approach to systematics to correctly delimit species (e.g., Schlick-Steiner et al. 2010), a conclusive application of characters other than morphological is often impossible for the type materials. Integrative approaches are also hindered by lack of any alternative data for the type species in a genus, or for any other more closely related species, whatsoever. Our efforts to integratively describe the species presented here were unsuccessful. Nevertheless, we argue this is the way to move forward in the taxonomic study of Ecuadorian sponges, and beyond this, we recommend sponge taxonomists to turn integrative taxonomy into routine work, so, as a community, we can build a reference dataset to help determine the true extent of relatedness among sponge lineages.

Ecuador is a rare, if not unique, example of nation where 97% (87 of 90 recorded species) of the knowledge of the poriferan biological resource, mainly composed of Demospongiae, is derived from an offshore location (Schuster et al. 2018, van Soest et al. 2020). Irrespective of the biological importance of the Galapagos Archipelago, this fact needs a major reversal. It is doubtful that there will ever be a proliferation of research institutes in the archipelago that might deepen the study of several aspects of these holobionts, whose transfer to mainland Ecuador is unlikely, at the least. Getting to know more the easily accessible sponges from the coast of mainland Ecuador is essential to develop this scientific field in the country, as highlighted from pioneering bioprospecting conducted at Reserva Marina El Pelado (Calabro et al. 2018).

The rather localized distributional data generated in this study does not permit to establish biogeographic boundaries for marine sponges along the Ecuadorian mainland. These will ultimately depend on expanding the taxonomic inventory to additional localities to the S and N of MPA El Pelado. Nevertheless, present data help filling in an important knowledge gap on the distribution of Tropical Eastern Pacific sponges, by expanding the notoriously underestimated sponge biodiversity inventory into the Guayaquil Ecoregion. It is expected that sponges will produce patterns similar to those recently reported for Ecuadorian zoantharians (Jaramillo et al. 2018), where Panamanian and Humboldtian affinities are clearly evident, indicating species coming from the north, and from the south, respectively. The first of these patterns is apparent for C. aff. californica, known all the way up to Mexico, at least. However, species illustrating affinities to the colder South American Pacific, as well as to the Galapagos Islands remain to be spotted on coastal mainland Ecuador marine sponges.

## Supplementary Material

XML Treatment for Tedania (Tedania) ecuadoriensis

XML Treatment for Cliona aff. euryphylle

XML Treatment for Subgenus Callyspongia

XML Treatment for Callyspongia (Callyspongia) aff.californica
